# Fluoroquinolone prescribing to older adults following FDA boxed warnings: a comparative analysis

**DOI:** 10.1017/ash.2026.10399

**Published:** 2026-05-05

**Authors:** Miaoqing Jia, James Flory, Emma E. McGinty, Tony Rosen, Yongkang Zhang

**Affiliations:** 1 Weill Cornell Medicine, USA; 2 Memorial Sloan Kettering Cancer Center, USA

## Introduction

Fluoroquinolones are broad spectrum antibiotics used to treat respiratory tract, urinary tract, and skin infections. Fluoroquinolone use is associated with serious adverse events among older adults, including tendinopathy, peripheral neuropathy, and aortic dissection.^1^ The Food and Drug Administration (FDA) has issued successive boxed warnings on fluoroquinolones for peripheral neuropathy (2013), restricting use for uncomplicated acute bacterial sinusitis, chronic bronchitis exacerbations, urinary tract infections due to unfavorable risk-benefit profiles (2016); and for increased risk of aortic aneurysm and dissection (2018).^2^ A study found overall decline in prescribing of fluoroquinolones after the 2013 and 2016 FDA warnings.^3^ However, this study did not account for broader changes in antibiotic prescribing, particularly antimicrobial stewardship programs, which optimize antibiotic use across care settings and expanded rapidly after the Centers for Disease Control and Prevention (CDC) released Core Elements in 2014 and Centers for Medicare and Medicaid Services (CMS) mandated these programs for hospitals in 2020.^4^ To date, there is little evidence on whether FDA warnings have effectively reduced fluoroquinolone use among older adults. To address this gap, we compared fluoroquinolone prescribing to macrolides as the primary comparator and cephalosporins and penicillins as the secondary comparators, selected based on overlapping clinical indications with fluoroquinolones. None of these comparators received new warnings during the study period.

## Methods

In this retrospective cohort study, we used the CMS Medicare Part D Prescriber Public Use Files from 2013 to 2023 containing provider-level prescription data for beneficiaries aged ≥65 years.^5^ We identified fluoroquinolone (ciprofloxacin, levofloxacin, moxifloxacin, ofloxacin, gemifloxacin), macrolide (azithromycin, clarithromycin, erythromycin), cephalosporin (32 medications), and penicillin (8 medications) prescriptions using generic drug names, restricted to systemic formulations. All drug names are provided in Supplementary Material. Nitrofurantoin was not included because it is indicated only for uncomplicated urinary tract infections, not for the respiratory and skin indications examined in this study. We calculated annual prescribing rates as the mean of provider-level rates for each antibiotic class. We conducted a comparative interrupted time series analysis with macrolides as the primary comparator and 2016 as the intervention point because the July 2016 warning was the first to restrict fluoroquinolone prescribing for specific infections. We estimated a segmented linear regression model with indicator variables for drug class, time, post 2016 period, and all interaction terms. The three-way interaction term estimates the difference-in-differences (DiD) coefficient, measuring whether fluoroquinolone prescribing trends changed differently than comparator trends after 2016. We used robust standard errors and tested parallel trends. Sensitivity analyses used cephalosporins and penicillins as additional comparators. Sulfonamides were also originally assessed but excluded from interpretation due to evidence of non-parallel trends. We conducted a sensitivity analysis to address potential impact of the COVID-19 on the results by excluding 2020–2021 from the full study period. The DiD design controls for secular trends affecting all antibiotic classes, including unmeasured stewardship adoption. Nurse practitioners were included as primary care providers consistent with prior evidence in this Medicare population.^6^ The Weill Cornell Medicine Institutional Review Board approved this study with a waiver of consent because it used publicly available, de-identified data. Additional methodological details are in the Supplementary Material.

## Results

**Figure 1.**
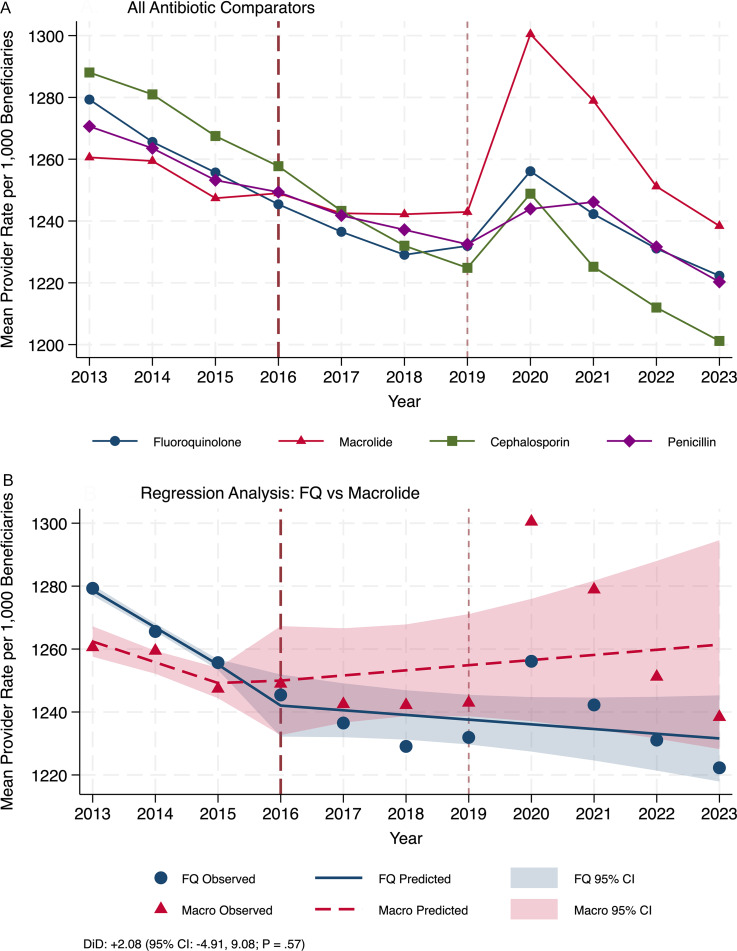
Antibiotic prescribing trends and comparative interrupted time series analysis, 2013–2023. Panel A shows prescribing rates per 1,000 Medicare beneficiaries (mean of provider-level rates) for four antibiotic classes. Circles: fluoroquinolones; triangles: macrolides; squares: cephalosporins; diamonds: penicillins. All classes showed declining trends before 2016 that attenuated after 2016. Panel B shows observed rates (points) and predicted values from segmented linear regression (lines) with 95% confidence intervals (shaded bands) for fluoroquinolones and macrolides. Pre-2016 trends were parallel (*P* = .16). The difference-in-differences coefficient was +2.08 (95% CI, −4.91 to 9.08; *P* = .57), indicating no differential effect. Vertical dashed line indicates the July 2016 FDA boxed warning (the first to restrict prescribing for specific infections); dotted line indicates the December 2018 warning for aortic dissection. Sensitivity analyses comparing fluoroquinolones to cephalosporins and penicillins showed fluoroquinolones declined less steeply after 2016 (Supplementary Table S2). Source: Centers for Medicare and Medicaid Services Part D Prescriber Public Use Files.

This study included 4,849,566 provider-year observations. All four antibiotic classes showed declining trends before 2016 (Figure 1A). From 2013 to 2015, mean provider-level rates declined by 11.8 per 1,000 beneficiaries annually for fluoroquinolones (from 1,279 to 1,256), by 6.6 annually for macrolides (from 1,261 to 1,247), and similarly for cephalosporins and penicillins. After 2016, all classes showed attenuated declines. In the primary comparative analysis, the change in fluoroquinolone trends did not differ statistically from macrolide trends (DiD, +2.08; 95% CI, −4.91 to 9.08; *P* = .57) (Figure 1B), indicating warnings did not differentially affect fluoroquinolone prescribing beyond concurrent secular trends. By 2023, mean provider-level rates were 1,222 per 1,000 beneficiaries for fluoroquinolones and 1,238 for macrolides. Prewarning trends were parallel (*P* = .16). Secondary and sensitivity analyses showed similar results (Supplementary Tables S1–S2). Results of the sensitivity analysis excluding the COVID-19 period (2020–2021) were consistent with the primary analysis (Supplementary Table S4).

## Discussion

Declining fluoroquinolone prescribing among older adults likely reflects secular trends in antibiotic use driven by antimicrobial stewardship programs rather than the specific impact of FDA warnings, as demonstrated by parallel declines in macrolides (respiratory infections), cephalosporins, and penicillins (urinary tract infections). The parallel declines observed across antibiotic classes suggest that concurrent nationwide initiatives, most notably the expansion of antimicrobial stewardship programs, were important drivers of change.^8–10^ While prior research noted absolute declines in fluoroquinolone use following FDA warnings,^3,7^ our comparative analysis suggests that these warnings did not achieve a differential reduction beyond existing trends. This suggests that the FDA warning may have had limited incremental impact on fluoroquinolone prescribing if stewardship programs had already been reducing these high-risk medications.^10^ To reduce harms from fluoroquinolones and similar high-risk medications with serious adverse effects, warnings may be more effective when coupled with stewardship strategies that support changes in prescribing practice, such as provider education, evidence-based guideline dissemination, and prospective audit and feedback.^10^

Limitations include provider-level aggregated data that prevented individual patient assessment, precluded examination of patient demographics including age, sex, or geography, and introduced possible duplicate counting if beneficiaries saw multiple providers. Annual data could not capture the mid-year timing of the July 2016 warning. The data do not specify clinical indication or route of administration, preventing restriction to indications targeted by the warning (acute bacterial sinusitis, bronchitis, and uncomplicated urinary tract infections) or distinction of oral from intravenous formulations.

## Supporting information

10.1017/ash.2026.10399.sm001 Jia et al. supplementary material Jia et al. supplementary material

## Data Availability

Research transparency and reproducibility. The data underlying this study are publicly available from the Centers for Medicare and Medicaid Services Medicare Part D Prescriber Public Use Files (https://data.cms.gov/provider-summary-by-type-of-service/medicare-part-d-prescribers). Analytical code is available from the corresponding author upon reasonable request.

## Supplementary material

The supplementary material for this article can be found at https://doi.org/10.1017/ash.2026.10399.
