# Supplementary material for: Fluoroquinolone prescribing to older adults following FDA boxed warnings: a comparative analysis
Source: Antimicrob Steward Healthc Epidemiol. 2026 May 5;6(1):e125. doi: 10.1017/ash.2026.10399 (PMC13150466; doi:10.1017/ash.2026.10399)
Supplement: Jia et al. supplementary material [file S2732494X26103994sup001.pdf]

Fluoroquinolone Prescribing to Older Adults Following FDA Boxed Warnings: A Comparative Analysis

Supplemental Materials

Supplementary Text S1. Detailed Statistical Methods

Study Design

We conducted a comparative interrupted time series analysis of fluoroquinolone prescribing to Medicare beneficiaries aged ≥ 65 years. We used macrolides as the primary comparison group and conducted sensitivity analyses with additional antibiotic comparators.

Data Source

Medicare Part D Prescriber Public Use Files from 2013 through 2023 contain provider-level prescription data for Medicare Part D beneficiaries. The files report total prescriptions, unique beneficiaries, provider specialty, and state for each provider-drug-year combination. Data are suppressed for combinations with fewer than 11 beneficiaries.

Data Limitations

The Medicare Part D data do not include diagnosis codes or route of administration. We could not restrict analyses to the three indications targeted by the July 2016 warning (acute bacterial sinusitis, bronchitis, uncomplicated urinary tract infections) or distinguish oral from other formulations. Our analysis includes all prescriptions for the identified antibiotic classes, including topical formulations of ofloxacin, which may marginally inflate fluoroquinolone counts; however, these represent a small fraction of total prescriptions. Erythromycin is also frequently prescribed as a prokinetic agent; however, this affects macrolide counts throughout the entire study period, introducing conservative rather than differential bias.

Drug Identification

We identified antibiotics using generic drug names in the Medicare files:

- **Fluoroquinolones:** ciprofloxacin, levofloxacin, moxifloxacin, ofloxacin, gemifloxacin
- **Macrolides:** azithromycin, clarithromycin, erythromycin
- **Cephalosporins:** cefaclor, cefadroxil, cefazolin, cefdinir, cefditoren, cefepime, cefixime, cefotaxime, cefotetan, cefoxitin, cefpodoxime, cefprozil, ceftaroline, ceftazidime, ceftibuten, ceftriaxone, cefuroxime, cephalexin, and others containing "cef" or "ceph" prefix
- **Penicillins:** penicillin, amoxicillin, ampicillin, amoxicillin-clavulanate, dicloxacillin, nafcillin, oxacillin, piperacillin
- **Sulfonamides:** sulfamethoxazole, trimethoprim, trimethoprim-sulfamethoxazole

Rationale for Comparator Selection

We selected macrolides as the primary comparator because they treat respiratory infections similar to fluoroquinolones in older adults. However, macrolides are not clinically appropriate for urinary tract infections, one of the three indications targeted by the July 2016 warning. We therefore conducted sensitivity analyses using cephalosporins and penicillins as additional comparators, as these antibiotic classes are prescribed for urinary tract infections. Sulfonamides were also originally assessed but excluded from analysis due to violation of the parallel trends assumption ($P = 0.006$).

Prescribing Rate Calculation

For each drug class and calendar year (2013-2023), we calculated rates in two steps:

First, we calculated provider level rates by dividing total prescriptions by total beneficiaries and multiplying by 1,000 for each provider.

$$\text{Provider Rate} = \frac{\text{Total Claims}}{\text{Total Beneficiaries}} \times 1000$$

Second, we calculated the national rate as the mean of provider level rates.

$$\text{National Rate}_{\text{drug, year}} = \frac{1}{N}\sum_{i=1}^{N} \text{Provider Rate}_{i,\text{drug,year}}$$

where *N* is the number of providers prescribing that drug class in that year.

Statistical Analysis

Primary Analysis: Comparative Interrupted Time Series Regression

We used interrupted time series regression with the following model:

$$\text{Rate}_{dt} = \beta_0 + \beta_1\text{FQ}_d + \beta_2\text{Time}_t + \beta_3\text{Post2016}_t + \beta_4(\text{FQ}_d \times \text{Time}_t) + \beta_5(\text{FQ}_d \times \text{Post2016}_t) + \beta_6(\text{Time}_t \times \text{Post2016}_t) + \beta_7(\text{FQ}_d \times \text{Time}_t \times \text{Post2016}_t) + \varepsilon_{dt}$$

where:

- Rate_{dt} = prescribing rate for drug *d* in year *t*
- FQ_d= indicator for fluoroquinolone (1) vs. macrolide (0)
- Time_t= years since 2015 (centered at baseline year)
- $Post2016_t$= indicator for years ≥2016
- ε_{dt} = error term

We used heteroskedasticity-robust standard errors (Huber-White) to account for potential heterogeneity in variance across observations.

Coefficient Interpretation:

The key parameter is β_7, which estimates whether the change in fluoroquinolone prescribing trends after 2016 differs from the change in macrolide prescribing trends. This DiD coefficient isolates the FDA warning effect from secular trends affecting both antibiotics.

Parallel Trends Assumption

The validity of DiD estimation requires that fluoroquinolones and macrolides would have followed parallel trends in the absence of FDA warnings ("parallel trends assumption"). We tested this assumption by examining pre-intervention trends (2013-2015):

$$\text{Rate}_{dt} = \gamma_0 + \gamma_1\text{FQ}_d + \gamma_2\text{Time}_t + \gamma_3(\text{FQ}_d \times \text{Time}_t) + \varepsilon_{dt}\text{Rate}_{dt}$$

for observations where $\text{Post2016} = 0$.

We tested H_0: $\gamma_3 = 0$ using an F-test. Failure to reject the null hypothesis ($P > .05$) supports the parallel trends assumption, indicating that pre-intervention trends did not differ significantly between drug classes.

Sample Size and Statistical Power

The primary analysis includes 22 national-level observations (2 drug classes × 11 years). The comparative design increases efficiency by doubling the effective sample size compared to single-group interrupted time series. Post-hoc power analysis indicates 80% power to detect effect sizes of approximately ±8 mean provider-level rate per 1,000 per year at α=0.05.

Sensitivity Analyses

Primary Care Providers

To address potential concerns about beneficiaries seeing multiple providers (leading to duplicate counting in provider-level data), we conducted a sensitivity analysis restricted to primary care providers (Family Practice, Internal Medicine, General Practice, Geriatric Medicine, Nurse Practitioner). These provider types account for the majority of antibiotic prescribing and represent patients' principal source of care, minimizing potential overlap from specialist consultations. We repeated the full comparative ITS analysis using only these providers.

Multiple Antibiotic Comparators

We conducted separate comparative interrupted time series analyses comparing fluoroquinolones to cephalosporins and penicillins using methods identical to the primary macrolide analysis.

COVID-19 Pandemic Sensitivity Analyses

To assess whether COVID-19 pandemic affected our primary results, we conducted a sensitivity analysis excluding 2020-2021 from the full study period. The analytic approach was identical to the primary analysis.

Total Antibiotic Prescribing

To examine whether secular trends reflected substitution between classes or reduced overall prescribing, we calculated total prescribing across all five antibiotic classes for each year.

Software

All analyses were conducted using Stata 18.0. Code is available from the authors upon request.

Supplementary Text S2. Detailed Results

Trend Decomposition

To aid interpretation, we decomposed trends into pre- and post-2016 components for each antibiotic class using simple linear regression within each period:

Fluoroquinolones:

- Pre-2016 trend: -11.8 mean provider-level rate per 1,000 per year (95% CI: -21.7 to -1.9; P=.042)
- Post-2016 trend: -1.5 mean provider-level rate per 1,000 per year (95% CI: -4.3 to 1.4; P=.248)
- **Trend change: +10.3 mean provider-level rate per 1,000 per year**

Macrolides:

- Pre-2016 trend: -6.6 mean provider-level rate per 1,000 per year (95% CI: -34.9 to 21.7; P=.207)
- Post-2016 trend: +1.6 mean provider-level rate per 1,000 per year (95% CI: -4.8 to 8.1; P=.560)
- **Trend change: +8.2 mean provider-level rate per 1,000 per year**

Differential trend change (fluoroquinolones vs. macrolides): +2.1 mean provider-level rate per 1,000 per year (P=.57)

This decomposition shows that both antibiotic classes experienced similar attenuations in declining trends after 2016, with no significant differential change.

Multiple Comparator Results

Parallel trends tests showed no differential pre-2016 trends for macrolides (P=0.16), cephalosporins (P=0.42), or penicillins (P=0.09). Due to evidence of non-parallel trends (P=0.006), sulfonamides were excluded from analysis.

DiD analysis showed no differential effect for macrolides:

- Macrolides: +2.08 (95% CI: -4.91 to 9.08; P=0.57)

Compared to cephalosporins and penicillins, fluoroquinolones declined less steeply after 2016:

- Cephalosporins: +6.56 (95% CI: 2.70 to 10.42; P=0.005)
- Penicillins: +4.17 (95% CI: 0.55 to 7.79; P=0.04)

These positive coefficients indicate that fluoroquinolone prescribing held up better than these comparators after 2016, further supporting the absence of a warning-specific effect on fluoroquinolone prescribing.

COVID-19 Pandemic Sensitivity Analysis Results.

In the sensitivity analysis excluding 2020–2021, difference-in-differences coefficients remained positive for all comparators (Supplementary Table S4). The macrolide comparison yielded a DiD of +3.22 (95% CI: −1.87 to 8.31; P = .190); cephalosporins +6.44 (95% CI: 2.73 to 10.14; P = .003); and penicillins +4.11 (95% CI: 1.05 to 7.17; P = .014). These results indicate that elevated macrolide use during the pandemic did not bias the comparator trend in a direction that would mask a warning-specific fluoroquinolone effect.

Supplementary Table S1. Primary Care Provider Sensitivity Analysis

Sample: Family Practice, Internal Medicine, General Practice, Geriatric Medicine, Nurse Practitioners (64.5% of observations)

Analysis	N	DiD Coefficient	95% CI	P-value	R^2
All Providers	22	+2.08	-4.91, 9.08	0.57	0.439
Primary Care Only	22	+4.29	-1.40, 9.98	0.16	0.753

Parallel Trends Test (Primary Care): $F(1, 2) = 3.98$, $P = 0.184$

Results were consistent across provider types, with parallel trends assumptions satisfied in both analyses.

Supplementary Table S2. Sensitivity Analyses: Multiple Antibiotic Comparators

Comparator	2015 Rate*	2023 Rate*	Parallel Trends P†	DiD Coefficient‡	95% CI	P-value
Macrolide (primary)	1,247	1,238	0.16	+2.08	-4.91 to 9.08	0.57
Cephalosporin	1,268	1,201	0.42	+6.56	2.70 to 10.42	0.005
Penicillin	1,253	1,220	0.09	+4.17	0.55 to 7.79	0.04
Sulfonamide§	—	—	0.006	—	—	—

* Total mean provider-level rate per 1,000 Medicare beneficiaries aged ≥65 years
†P-value for pre-2016 interaction term testing parallel trends assumption
‡DiD coefficient; positive values indicate fluoroquinolone prescribing declined less than comparator
§ Evidence of non-parallel trends suggests potential violation of the parallel trends assumption; excluded from analysis. DiD coefficient not estimated.

Positive significant coefficients for cephalosporins and penicillins indicate fluoroquinolone prescribing declined less steeply than these comparators after 2016, supporting the conclusion that observed fluoroquinolone trends reflected secular changes rather than warning-specific effects

Supplementary Table S3. Total Antibiotic Prescribing, 2013-2023

Year	Total Prescriptions*	Change from 2015
2013	3,688	+7.6%
2014	3,593	+4.8%
2015	3,429	Reference
2016	3,377	-1.5%
2017	3,266	-4.7%
2018	3,212	-6.3%
2019	3,075	-10.3%
2020	3,477	+1.4%
2021	3,233	-5.7%
2022	3,066	-10.6%
2023	3,025	-11.8%

* Total mean provider-level rate per 1,000 beneficiaries across fluoroquinolones, macrolides, cephalosporins, penicillins, and sulfonamides

Total antibiotic prescribing declined 12% from 2015 to 2023, consistent with both substitution between antibiotic classes and reduced unnecessary prescribing from antibiotic stewardship efforts. The increase in 2020 likely reflects COVID-19 pandemic effects on healthcare utilization and prescribing patterns.

Supplementary Table S4. COVID-19 Pandemic Sensitivity Analyses: Difference-in-Differences Coefficients

Analysis	Comparator	Parallel Trends P*	DiD Coefficient†	95% CI	P-value
Excluding 2020	Macrolide	0.16	+3.22	−1.87, 8.31	0.190
	Cephalosporin	0.42	+6.44	2.73, 10.14	0.003
	Penicillin	0.09	+4.11	1.05, 7.17	0.014

* P-value for pre-intervention interaction term testing parallel trends assumption; all satisfy parallel trends (P > .05)
† DiD coefficient from segmented linear regression (prescriptions per 1,000 Medicare beneficiaries per year); positive values indicate fluoroquinolone prescribing declined less steeply than the comparator

Positive coefficients for all comparators indicate fluoroquinolone prescribing declined less steeply than comparators after 2016, inconsistent with a warning-specific effect; the macrolide comparison was non-significant, consistent with the primary analysis.

Supplementary Figure S1. Antibiotic Prescribing as Percentage of Total

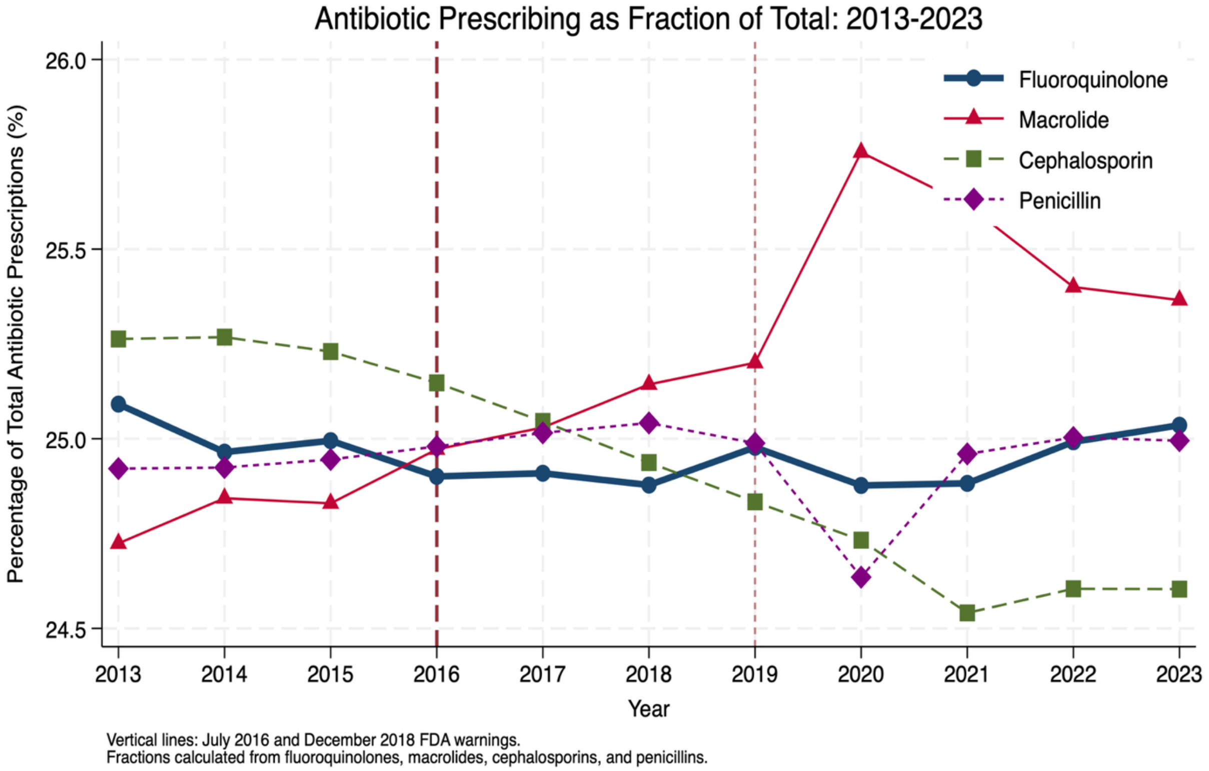

Each line represents the percentage of total antibiotic prescriptions for each class among Medicare beneficiaries aged ≥65 years. Circles: fluoroquinolones; triangles: macrolides; squares: cephalosporins; diamonds: penicillins. Vertical dashed line indicates the July 2016 FDA boxed warning; dotted line indicates the December 2018 warning. Fluoroquinolone market share remained relatively stable throughout the study period (range: 23-25%), consistent with the finding that FDA warnings did not differentially reduce fluoroquinolone prescribing. The stable proportions across all classes support the conclusion that observed trends reflected secular changes affecting all antibiotics rather than warning-specific effects.
Source: Centers for Medicare and Medicaid Services Part D Prescriber Public Use Files.

Supplementary Figure S2. Primary Antibiotic Classes, 2013-2023

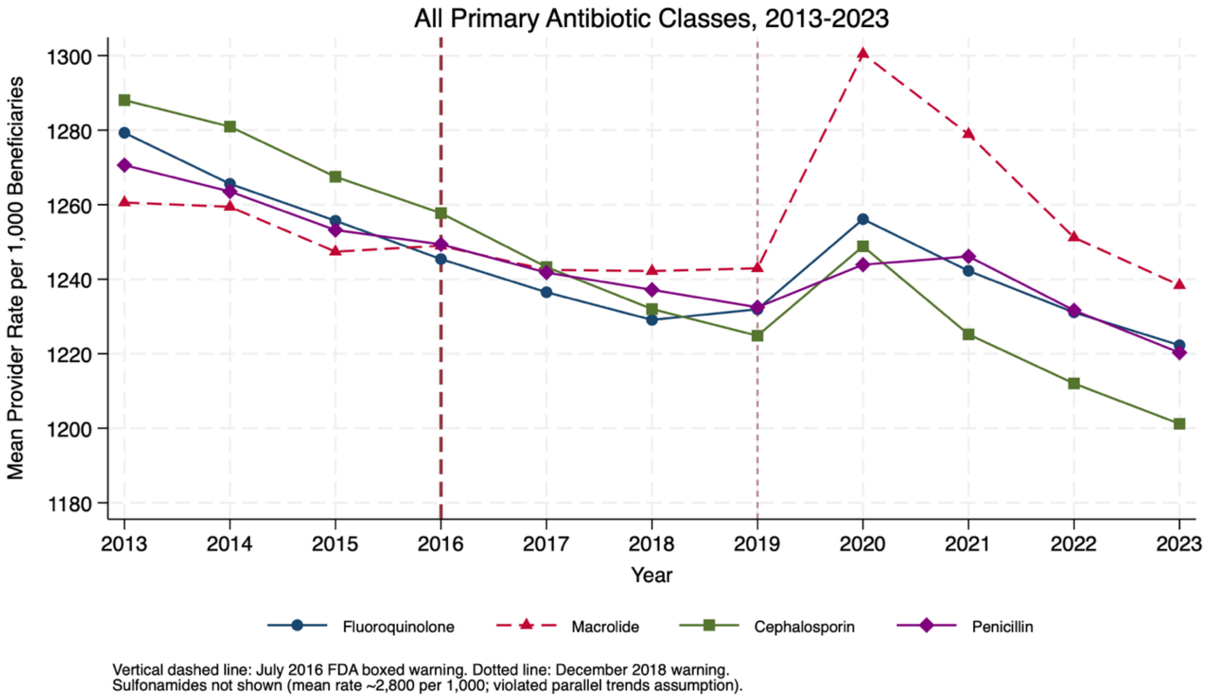

Total mean provider-level prescribing rate per 1,000 Medicare beneficiaries aged ≥65 years for fluoroquinolones, macrolides, cephalosporins, and penicillins, 2013–2023. Circles: fluoroquinolones; triangles: macrolides; squares: cephalosporins; diamonds: penicillins. Vertical dashed line indicates the July 2016 FDA boxed warning; dotted line indicates the December 2018 warning.
Source: Centers for Medicare and Medicaid Services Part D Prescriber Public Use Files.

Supplementary Figure S3. Total Antibiotic Prescribing

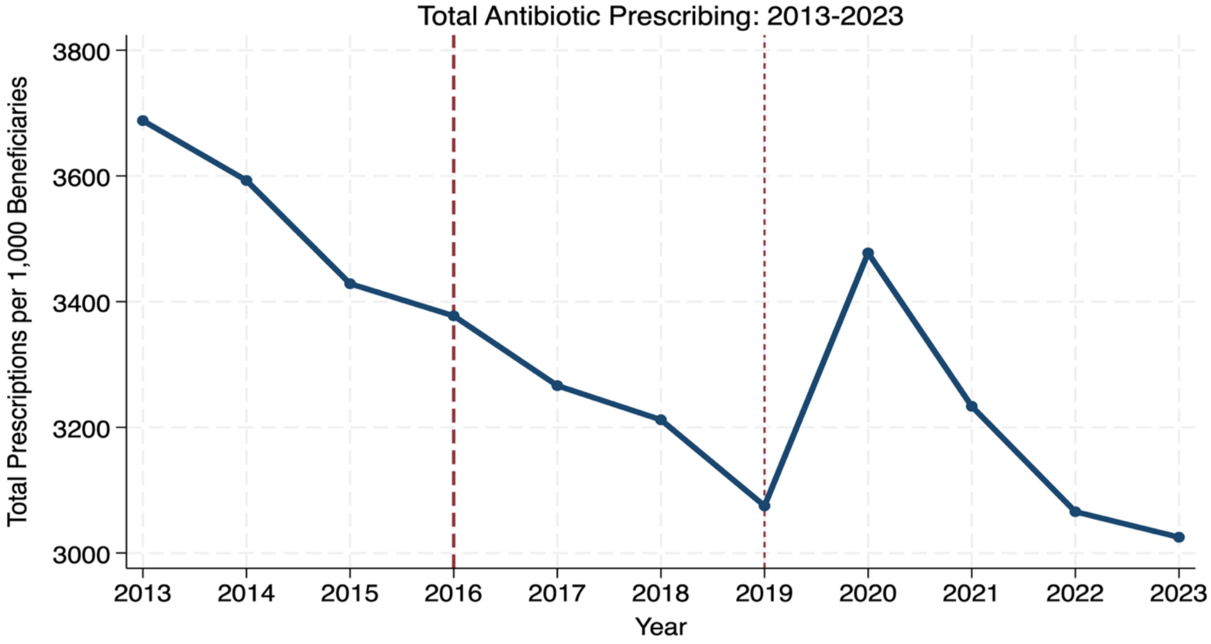

Total mean provider-level rate per 1,000 Medicare beneficiaries across fluoroquinolones, macrolides, cephalosporins, penicillins, and sulfonamides, 2013–2023. Sulfonamides are included for descriptive completeness only and were excluded from all comparative analyses due to violation of the parallel trends assumption. Vertical dashed line indicates the July 2016 FDA boxed warning; dotted line indicates the December 2018 warning. Total antibiotic prescribing declined from 3,688 per 1,000 in 2013 to 3,025 in 2023 (−18.0%), with a temporary increase in 2020 (3,477 per 1,000) likely related to COVID-19 pandemic effects, consistent with secular declines driven by antimicrobial stewardship efforts.
Source: Centers for Medicare and Medicaid Services Part D Prescriber Public Use Files.

Supplementary Figure S4. COVID-19 Sensitivity Analysis: Fluoroquinolone vs Macrolide Prescribing.

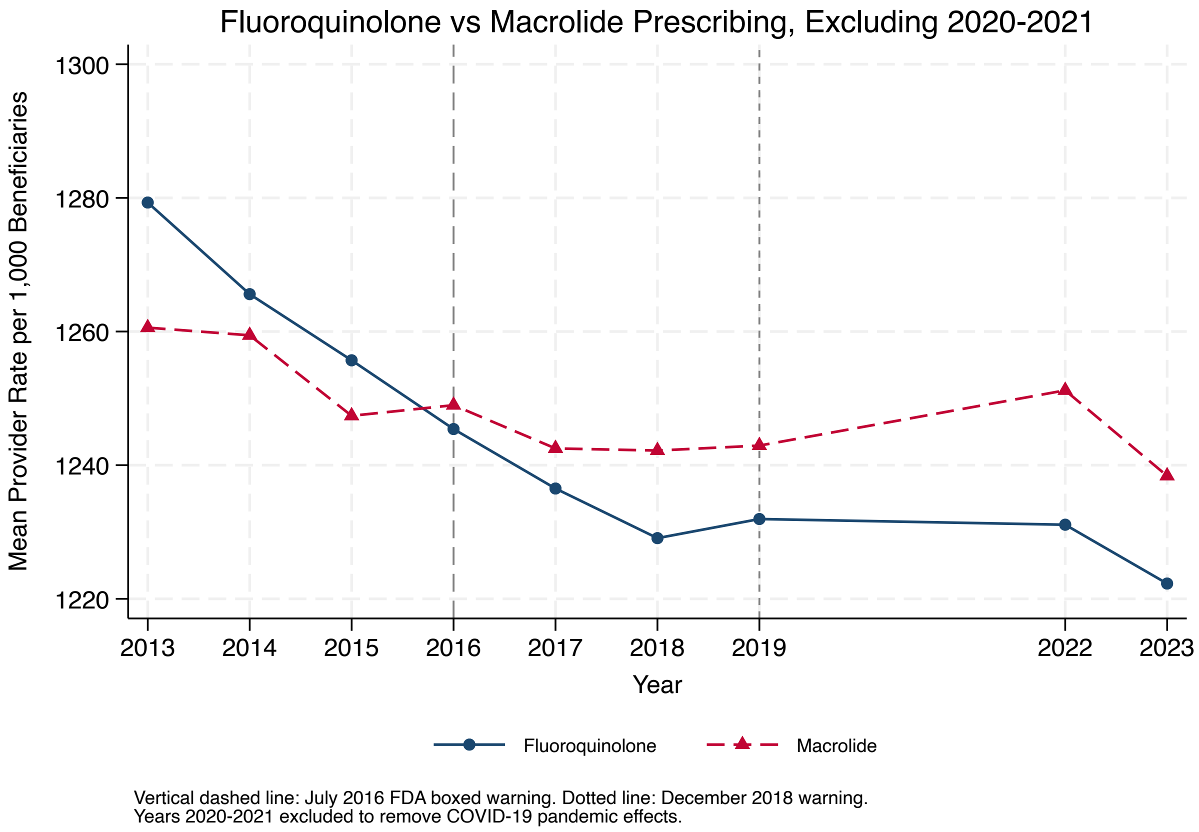

Observed prescribing rates per 1,000 Medicare beneficiaries for fluoroquinolones (circles, solid line) and macrolides (triangles, dashed line), 2013–2023 with 2020–2021 excluded. The gap in the x-axis indicates the omitted pandemic years. Vertical dashed line indicates the July 2016 FDA boxed warning; dotted line indicates the December 2018 warning. Parallel declining trends are visible across both windows, with no evidence of differential fluoroquinolone-specific decline.
Source: Centers for Medicare and Medicaid Services Part D Prescriber Public Use Files.
